# Fish mitigate trophic depletion in marine cave ecosystems

**DOI:** 10.1038/s41598-018-27491-1

**Published:** 2018-06-15

**Authors:** Simona Bussotti, Antonio Di Franco, Carlo Nike Bianchi, Pierre Chevaldonné, Lea Egea, Emanuela Fanelli, Christophe Lejeusne, Luigi Musco, Carlos Navarro-Barranco, Alexis Pey, Serge Planes, Jean Vincent Vieux-Ingrassia, Paolo Guidetti

**Affiliations:** 1 Université Côte d’Azur, CNRS, FRE 3729 ECOMERS, Avenue Valrose 28, 06108 Nice, France; 2 CoNISMa, Piazzale Flaminio 9, 00196 Rome, Italy; 3 DiSTAV (Department of Earth, Environment and Life Sciences), University of Genoa, Corso Europa, 26, 16132 Genoa, Italy; 4 Institut Méditerranéen de Biodiversité et d’Ecologie Marine et Continentale (IMBE), UMR 7263 CNRS, IRD, Aix Marseille Université, Avignon Université, Station Marine d’Endoume, Marseille, France; 5 DiSVA (Department of Life and Environmental Sciences), Polytechnic University of Marche, Via Brecce Bianche, 60131 Ancona, Italy; 6 Sorbonne Université, CNRS. Station Biologique de Roscoff, UMR 7144 AD2M. Place Georges Teissier, 29688 Roscoff, France; 7 Stazione Zoologica Anton Dohrn-Integrative Marine Ecology Department, Villa Comunale, 80121 Naples, Italy; 8 Unidad de Zoología, Departamento de Biología, Universidad Autónoma de Madrid, Calle Darwin, 28049 Madrid, Spain; 9 PSL Research University: EPHE-UPVD-CNRS, USR 3278 CRIOBE, Université de Perpignan, 58 Avenue Paul Alduy, 66860 Perpignan, France; 10 Laboratoire d’Excellence «CORAIL», Polynésie, France

## Abstract

Dark marine habitats are often characterized by a food-limited condition. Peculiar dark habitats include marine caves, characterized by the absence of light and limited water flow, which lead to reduced fluxes of organic matter for cave-dwelling organisms. We investigated whether the most abundant and common cave-dwelling fish *Apogon imberbis* has the potential to play the role of trophic vector in Mediterranean marine caves. We first analysed stomach contents to check whether repletion changes according to a nycthemeral cycle. We then identified the prey items, to see whether they belong to species associated with cave habitats or not. Finally, we assessed whether *A. imberbis* moves outside marine caves at night to feed, by collecting visual census data on *A. imberbis* density both inside and outside caves, by day and by night. The stomach repletion of individuals sampled early in the morning was significantly higher than later in the day. Most prey were typical of habitats other than caves. *A. imberbis* was on average more abundant within caves during the day and outside during the night. Our study supports the hypothesis regarding the crucial trophic role of *A. imberbis* in connecting Mediterranean marine caves with external habitats.

## Introduction

Dark marine habitats are environments where the light is limited if not completely absent (aphotic areas), leading to a consequent shortage or total lack of autochthonous photosynthesis^1^. Setting aside chemosynthetic processes in deep ocean ridges and similar habitats^2^, the low/absent autochthonous primary production makes dark habitats food-limited environments. Their functioning, therefore, depends (partially or totally) on allochthonous primary production^3^. The vertical flux of faecal pellets, cadavers and, more generally, marine snow (i.e. the continuous fall of mostly organic detritus from the upper to the deeper layers) is an essential food source for organisms living in aphotic conditions^4,5^.

Underwater marine caves are particular dark habitats, quite common in shallow coastal rocky areas worldwide^6^. Inside, the light is significantly reduced if not completely absent, leading to ecological conditions comparable to the oligophotic/aphotic deep sea^7–9^. Light penetration within caves depends on the depth where the cave is located, and on internal cave shape and structure (e.g. size and number of openings, lateral branches, total length). In shady or dark cave sectors, the photo-autotrophic primary production is absent, with chemo-lithoautotrophic production occurring only in limited cases^10^.

Marine caves are common in the Mediterranean Sea, especially in karstic areas^11–13^. The extremely variable morphology of caves, from simple linear to complex shapes, is reflected in a diversity of physical-chemical gradients and trophic conditions inside, and consequently exerts relevant effects on resident biota (e.g.^14–18^). Depending on cave morphology, water movement can more or less effectively transport Particulate Organic Matter (POM) from outside habitats into the cave, a process that may affect the assemblages living inside, e.g. in terms of abundance, cover, biomass, species composition and overall assemblage structure^19–22^. In consequence, the diversity of benthic fauna tends to become progressively impoverished from the entrance towards the inner parts, especially in blind caves (single opening) where light penetration and water circulation significantly decrease with the distance from the entrance (e.g.^20,23–25^). Light attenuation (leading to primary production decrease) combined with the hydrological confinement (leading to reduced organic input) induces trophic depletion, which is responsible for the significant decrease in biomass, for both epifauna and infauna, inside marine caves (e.g.^26,27^). The trophic depletion within caves is the result of the shortage of the total food input, but it is also associated with an impoverishment of its nutritional value (C/N ratio, complex/simple organic matter ratio, and pheophitin-chlorophyll ratio inside the cave^7^).

Mobile fauna in marine caves can however be rich, mostly composed of teleost fishes and crustaceans^25^. Mobile cave-dwelling organisms that migrate for feeding outside the caves trophically connect the caves with outside habitats. This is done by transferring organic matter from nutrient-rich systems outside the caves to the nutrient-poor system when they get back into the caves^28^. Thus, it has been hypothesized that the nycthemeral migrations of mobile species could mitigate the trophic depletion of marine caves^25,26,29,30^. Caves, clearly, are not closed ecosystems and their ecological functioning includes the connectivity between inside and outside, via the active movement of mobile organisms. Some studies highlighted the important role of mysid crustaceans in marine caves through their faecal pellet production (e.g.^31,32^). The predation on mysids by sessile and mobile cave-dwelling predators emphasizes their role in energy transfer from the open waters to cave ecosystems^33,34^. In contrast, no study has investigated whether fish can have such a role. Fish are an important component of marine cave ecosystems, in terms of species richness, abundance and biomass^15,22,35–38^, and they could play a role as vectors of POM in marine caves.

**Figure 1:**
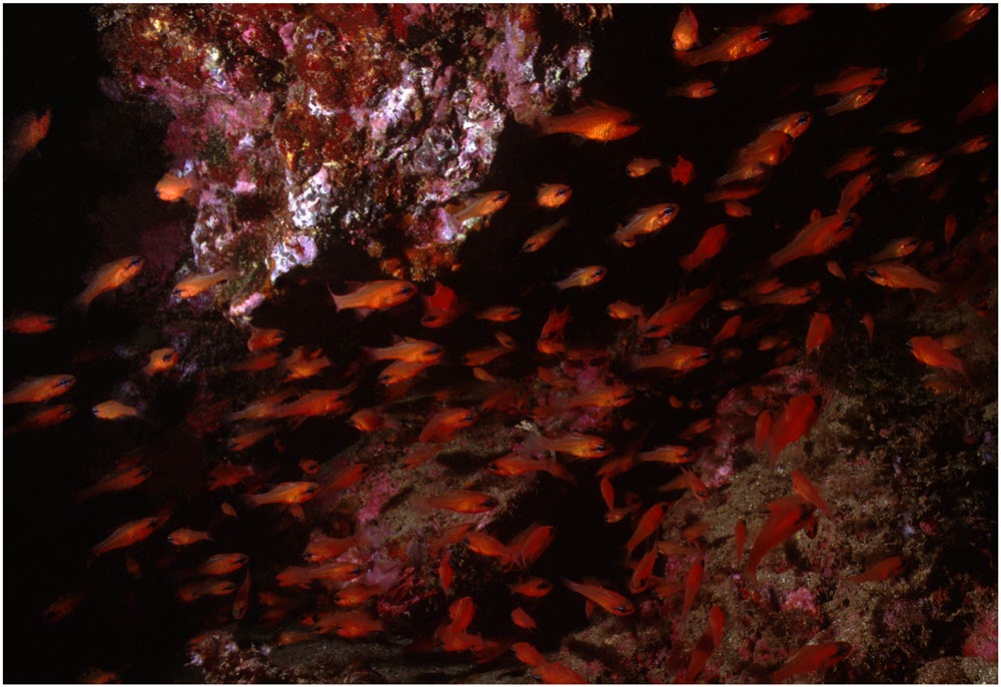
School of *Apogon imberbis* at the entrance of a marine cave.

In systems other than caves, a number of fishes, due to their day/night feeding migration and gregarious behaviour, have been described as nutrient-enriching organisms, significantly importing organic matter from nutrient-rich to nutrient-poor systems^39^. This role has been observed for the pomacentrid *Chromis punctipinnis* in rocky temperate habitats^40,41^ and for haemulid fishes in coral reefs^39^. In the Mediterranean Sea, the gregarious damselfish *Chromis chromis*^42^ is reported to feed in daytime on plankton in the water column, to then release its faeces, that are rapidly consumed by benthic detritivores, while resting at night in shelters close to the rocky substrate or seagrasses. As an example of darker habitats, diel vertical migrations of mesopelagic fishes (mostly the species of the family Myctophidae and the order Stomiiformes) strengthen the biological pump that transports organic carbon between surface and deeper waters^43,44^. A similar functional process could occur in marine caves. Recent data suggest that the cardinal fish *Apogon imberbis* (Apogonidae) may have the potential to enrich Mediterranean marine caves with POM^37^. This fish has a wide distribution range (Mediterranean Sea and Eastern Atlantic, from Portugal and the Azores, southward to Morocco and the Gulf of Guinea; www.fishbase.org). It is a sciaphilous species thriving in a number of shady/dark habitats (e.g. crevices and under boulders in rocky habitats, *Posidonia oceanica* matte), but it is particularly frequent in marine caves, where it forms large schools (Fig. 1). Inside the caves it can account for up to 70% and 30% of total fish density and biomass, respectively^37^. Similarly to other apogonid fishes^45–47^, *A. imberbis* is site-attached^48^ and more active at night^48–50^. In tropical coral reefs, apogonid fishes spend most of the day amongst branching corals and in cryptic habitats, often forming dense aggregations^51^. However, they make rapid incursions into surrounding habitats (open waters, sandy bottoms and seagrass) at night for feeding, then come back to corals where they release their dejections. In this way they play a role of vectors of organic matter^52^. Although several authors (e.g.^22,29,30,37,53^) have suggested a similar role for *A. imberbis* in marine caves (feeding outside the caves and releasing faeces inside), no attempt has ever been made to test this hypothesis. From this perspective, collecting information about the dietary patterns and nycthemeral movements of *A. imberbis* inhabiting Mediterranean marine caves is pivotal to better understanding its role and the way marine cave ecosystems function^22^. Information on the diet of cardinal fish is extremely scanty^49,54^, particularly on *A. imberbis* living in marine caves^34^.

**Figure 2:**
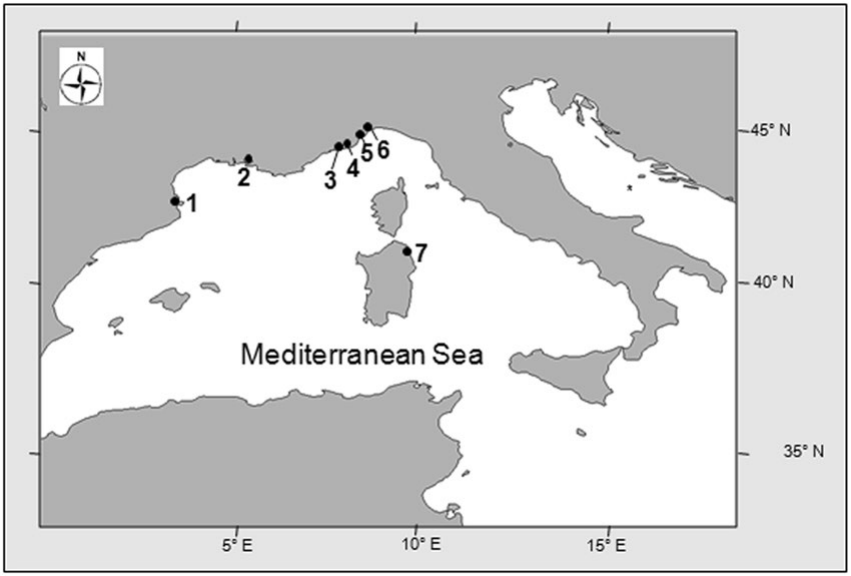
Map of the 7 areas where are located the 14 marine caves sampled to investigate feeding patterns of *Apogon imberbis*. The caves sampled for each area are listed in brackets: (1) Cap de Creus (Inferno, Pelegrì 1, Pelegrì 2); (2) Côte Bleue (La Vesse, Méjean, Niolon), (3) Villefranche-sur-Mer (Corail*°, Lido*°, Semaphore*); (4) Principality of Monaco (Grotte artificielle); (5) Ventimiglia (Secca); (6) Bergeggi (Bergeggi); (7) Tavolara (Ghigliottina, Papa). *°: caves sampled for *Apogon imberbis* gut content analyses (*) and for observations on foraging patterns (°). Map was generated using the open source QGis software version 2.6.1 (http://www.qgis.org/en/site/).

In the present study, we addressed three questions: (1) Do cave-dwelling *A. imberbis* feed at night? (2) Do prey items of cave-dwelling *A. imberbis* belong to taxa typically associated with habitats outside the caves? (3) Are there changes in the abundance patterns of *A. imberbis* within and outside caves during the night/day cycle? Positive answers to these questions would support the hypothesis that *A. imberbis* feeds outside caves at night to then release faecal pellet within caves during daytime. This would involve a transfer of organic matter from outside to the caves and a mitigation of their trophic depletion. Temporal patterns in the feeding behaviour of *A. imberbis* were assessed by combining information from stomach content analysis with the diurnal/nocturnal spatial distribution of the species (as a proxy of fish movement) inside and outside 14 marine caves located in the NW Mediterranean Sea (Spain, France, Monaco and Italy) (Fig. 2). To assess *A. imberbis* temporal patterns in feeding behaviour, we then focused on three caves few kilometres apart from each other within the same study area (Villefranche-sur-Mer, France). The objective was to get a finer taxonomical identification of the prey items of *A. imberbis* (to the species level whenever possible) in order to assess, on the basis of the known ecology of the prey taxa, whether the cave-dwelling *A. imberbis* feeds on prey living inside or outside cave habitats. Finally, we assessed in the same area, by means of underwater visual census, the spatio-temporal distribution of *A. imberbis* at day and night, inside and outside caves, also collecting *in situ* observations on its foraging behaviour.

## Results

### Feeding patterns

**Figure 3:**
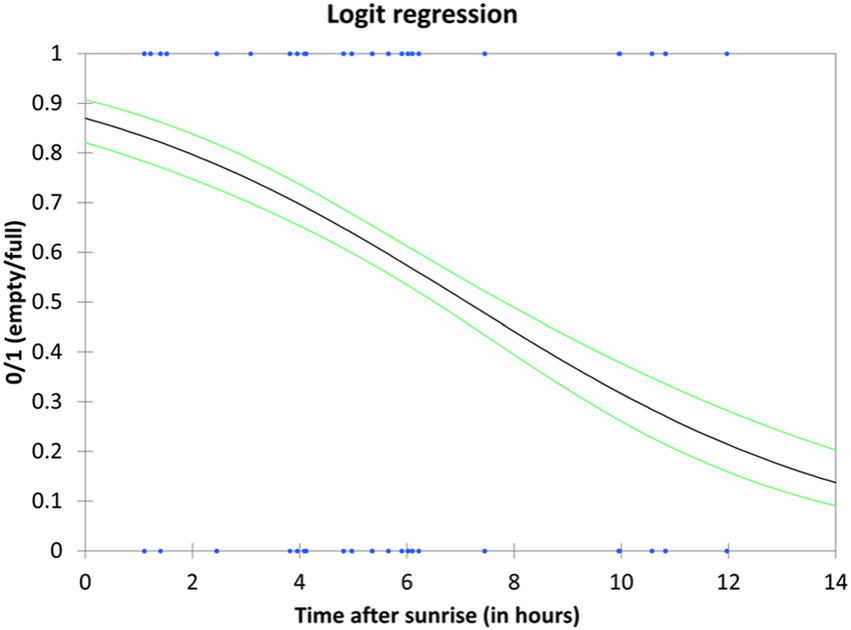
State of the *Apogon imberbis* stomach, expressed as empty/full according to time-interval between sunrise and time of sampling. 1 reflects full and 0 empty stomachs. Blue dots indicate observed data, black lines indicate predicted event probability following a logit regression model. Green curves indicate 95% confidence limits.

The logistic regression performed on the *Apogon imberbis* stomach repletion data from the 14 caves investigated shows a significant effect of the time-interval between sunrise and sampling time on the feeding pattern of *A. imberbis* (Wald = 86.930, df = 1, p < 0.01), with a significant decrease of stomach repletion with increasing time-interval (Fig. 3). Specifically, individuals sampled early in the morning (1–3 hours after sunrise) more frequently (~95%) had replete stomachs than individuals sampled later in the day (>9 hours after sunrise; <20%).

### Analysis of stomach contents

**Figure 4:**
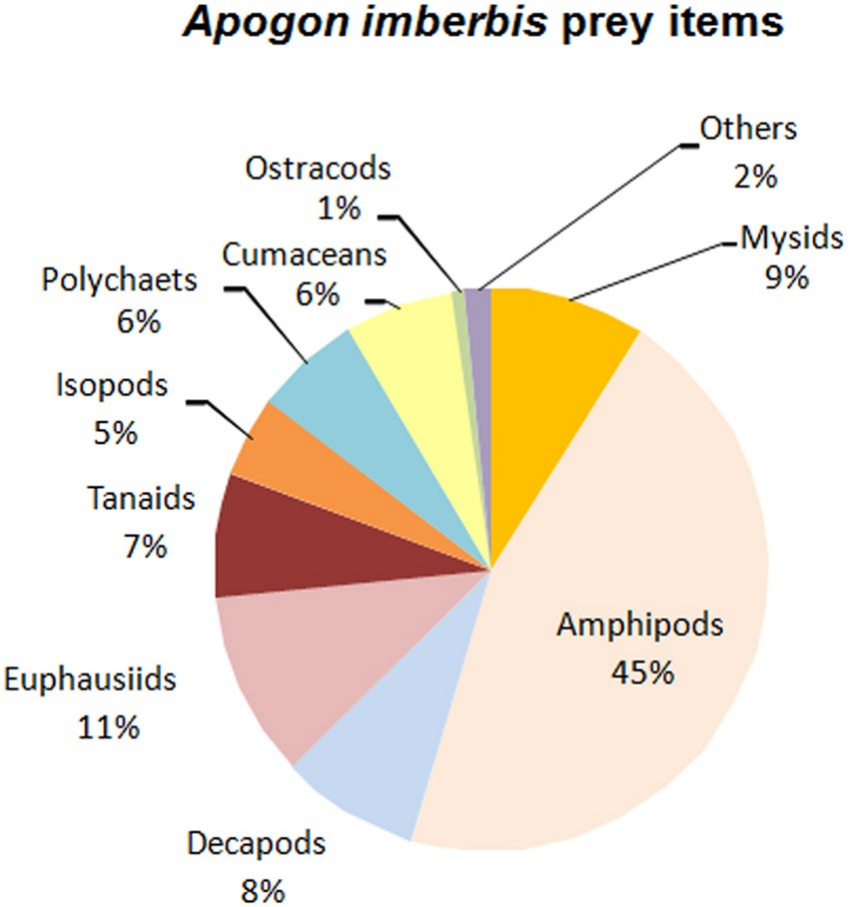
Percentage of the main prey groups ingested by *Apogon imberbis*.

A large variety of prey groups were found in the stomachs of *A. imberbis* sampled in the three caves of the Villefranche-sur-Mer, France area (Corail, Lido, Semaphore; Table 1; Fig. 4). Only in a very few cases the advanced state of digestion of the prey items prevented their identification. Considering the three caves together (Fig. 4), crustaceans strongly dominated the diet of *A. imberbis*, being present in 83% of the stomachs. Amphipods were by far the most strongly represented order (present in ~45% of stomachs), followed by euphausiids (11%), mysids (9%) and decapods (8%). Another important group, the annelids, was recorded in ~6% of the stomachs. A small fish otolith (species not identified) was found in just one case.

**Table 1:** Frequency of occurrence in percentage (FO%) and number of individuals of identifiable prey items (N, mean(se)) of *Apogon imberbis* in each of the three caves at Villefranche-sur-Mer.

Prey groups	GC		GL		GS	
	FO%	N	FO%	N	FO%	N
**Amphipoda**						
*Ampelisca* spp.	22.22	0.55 (0.21)	3.09	0.04 (0.03)	0.00	0 (NA)
*Ampithoe* sp.	0.00	0 (NA)	0.00	0 (NA)	1.96	0.02 (0.02)
*Apherusa* spp.	44.44	2.55 (0.85)	13.40	0.53 (0.19)	0.00	0 (NA)
*Aora* sp.	5.56	0.08 (0.06)	1.03	0.01 (0.01)	0.00	0 (NA)
*Atylus vedlomensis*	0.00	0 (NA)	1.03	0.01 (0.01)	0.00	0 (NA)
*Bathyporeia* sp.	0.00	0 (NA)	1.03	0.01 (0.01)	0.00	0 (NA)
*Caprella acanthifera*	22.22	0.33 (0.13)	4.12	0.05 (0.03)	1.96	0.02 (0.02)
*Dexamine spiniventris*	13.89	0.16 (0.07)	0.00	0 (NA)	0.00	0 (NA)
*Dexamine spinosa*	36.11	0.63 (0.16)	12.37	0.15 (0.04)	3.92	0.04 (0.03)
*Harpinia pectinata*	0.00	0 (NA)	1.03	0.01 (0.01)	0.00	0 (NA)
*Hippomedon massiliensis*	0.00	0 (NA)	3.09	0.03 (0.02)	0.00	0 (NA)
*Hyale* spp.	5.56	0.05 (0.03)	1.03	0.01 (0.01)	15.69	0.22 (0.08)
*Iphimedia* sp.	5.56	0.05 (0.03)	0.00	0 (NA)	0.00	0 (NA)
*Lembos* sp.	22.22	0.41 (0.15)	4.12	0.06 (0.03)	0.00	0 (NA)
*Leucothoe* sp.	2.78	0.02 (0.02)	0.00	0 (NA)	0.00	0 (NA)
*Liljeborgia* sp.	2.78	0.02 (0.02)	1.03	0.01 (0.01)	1.96	0.02 (0.02)
*Maera* sp.	0.00	0 (NA)	0.00	0 (NA)	7.84	0.16 (0.09)
*Microdeutopus* sp.	2.78	0.02 (0.02)	0.00	0 (NA)	0.00	0 (NA)
*Monoculodes* sp.	2.78	0.05 (0.05)	6.19	0.09 (0.04)	5.88	0.16 (0.10)
*Phtisica marina*	8.33	0.08 (0.04)	2.06	0.07 (0.06)	0.00	0 (NA)
*Stenothoe* sp.	0.00	0 (NA)	0.00	0 (NA)	1.96	0.02 (0.02)
Caprellidae (indet.)	8.33	0.08 (0.04)	4.12	0.04 (0.02)	1.96	0.04 (0.03)
Lysianassidae (indet.)	5.56	0.05 (0.03)	9.28	0.21 (0.14)	0.00	0 (NA)
**Decapoda**						
*Alpheus macrocheles*	2.78	0.02 (0.02)	0.00	0 (NA)	19.61	0.34 (0.08)
*Galathea bolivari*	0.00	0 (NA)	1.03	0.01 (0.01)	0.00	0 (NA)
*Liocarcinus navigator*	0.00	0 (NA)	1.03	0.01 (0.01)	0.00	0 (NA)
*Pagurus* spp.	0.00	0 (NA)	1.03	0.02 (0.02)	0.00	0.02 (0.02)
*Palaemon* sp.	0.00	0 (NA)	1.03	0.01 (0.01)	3.92	0.04 (0.03)
*Processa* sp.	0.00	0 (NA)	1.03	0.01 (0.01)	0.00	0 (NA)
*Eualus cranchii*	2.78	0.02 (0.02)	0.00	0 (NA)	0.00	0 (NA)
*Upogebia* sp.	2.78	0.02 (0.02)	1.03	0.01 (0.01)	0.00	0 (NA)
Gebiidea (indet.)	0.00	0 (NA)	0.00	0 (NA)	1.96	0.02 (0.02)
**Euphausiacea**						
*Meganychtiphanes norvegica*	2.78	0.02 (0.02)	0.00	0.00	0.00	0 (NA)
*Thysanoessa* sp.	2.78	0.91 (0.91)	6.19	0.42	0.00	0 (NA)
**Mysida**						
*Anchialina agilis*	2.78	0.02 (0.02)	1.03	0.03	0.00	0 (NA)
*Diamysis* sp.	0.00	0 (NA)	1.03	0.04	0.00	0 (NA)
*Erythrops* sp.	0.00	0 (NA)	3.09	0.06	0.00	0 (NA)
*Leptomysis* sp.	0.00	0 (NA)	8.25	0.18	0.00	0 (NA)
*Mysidopsis* sp.	0.00	0 (NA)	2.06	0.03	0.00	0 (NA)
*Siriella* sp.	2.78	0.02 (0.02)	1.03	0.01	1.96	0.02 (0.02)
**Annelida**						
*Eunice vittata*	0.00	0 (NA)	1.03	0.01	0.00	0 (NA)
*Lysidice ninetta*	0.00	0 (NA)	0.00	0.00	7.84	0.71 (0.36)
*Lysidice unicornis*	2.78	0.05 (0.05)	11.34	0.28	0.00	0 (NA)
*Perinereis oliveirae*	0.00	0 (NA)	1.03	0.01	0.00	0 (NA)
Eunicidae (indet.)	0.00	0 (NA)	2.06	0.02	0.00	0 (NA)
Opheliidae (indet.)	0.00	0 (NA)	2.06	0.02	0.00	0 (NA)

GC: Corail Cave (number of stomachs analyzed = 36); GL: Lido Cave (n = 97); GS: Semaphore Cave (n = 51). NA = not applicable.

The diet composition significantly differed among the three caves (PERMANOVA, pseudo-F: 6.206, p = 0.0001, Table 1). Among amphipods, *Dexamine spinosa*, *Apherusa* spp. and *Lembos* sp. were more frequent in the *A. imberbis* stomachs collected at Corail and Lido caves, while *Hyale* spp. and *Maera* spp. were found more frequently in the samples taken in Semaphore cave. Among the other crustaceans, the decapod *Alpheus macrocheles* was the most frequent prey at Semaphore cave. Euphausiacea, especially *Thysanoessa* sp., were exclusively found in samples taken at Corail and Lido caves, while mysids mainly prevailed in the stomachs of *A. imberbis* sampled at Lido cave. Finally, concerning polychaetes, *Lysidice unicornis* strongly dominated in the *A. imberbis* stomachs taken from Lido cave, while *Lysidice ninetta* was abundant and found just at Semaphore cave.

### Nycthemeral distribution patterns of *Apogon imberbis*

**Figure 5:**
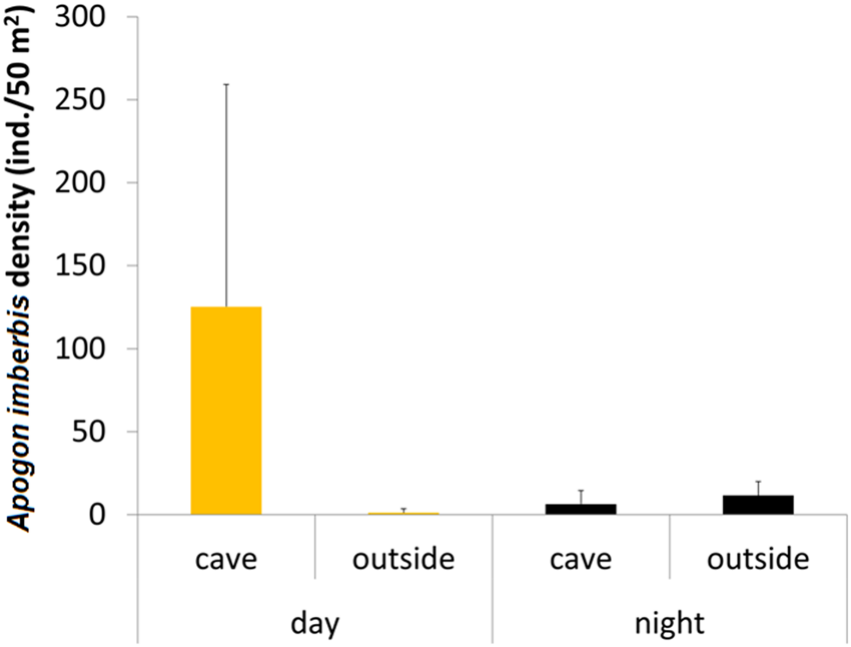
Density (mean ± standard error) of *Apogon imberbis* in the cave habitat and outside, by day and by night.

The analysis of the data obtained by carrying out underwater visual censuses performed by day and by night within and outside Corail and Lido caves showed that the density of *A. imberbis* during the day was on average 106 times higher inside the caves than outside (Fig. 5). At night, the pattern was the opposite (with a significant interaction between the factors ‘inside/outside habitat’ and ‘sampling time’; Table 2), with density outside the cave being on average twice as high as inside (Fig. 5). Overall, fish density at night decreased by 94.9% inside the cave, and increased by 894.5% outside.

**Table 2:** Results of univariate PERMANOVA analyses on *Apogon imberbis* density data.

Source	df	SS	MS	Pseudo-F	P(perm)	perms
Ha	1	667.03	667.03	69.882	**0.0155**	9952
DN	1	279.94	279.94	55.898	**0.0158**	9926
Si	1	0.23561	0.23561	9.5998E-2	0.7881	P(MC)
St	2	30.276	15.138	6.1678	0.1427	P(MC)
Ha × DN	1	1117	1117	50.986	**0.0075**	9929
Ha × Si	1	3.2548	3.2548	1.0213	0.4113	2100
Ha × St	2	12.672	6.3358	1.9881	0.3411	9953
DN × Si	1	0.67926	0.67926	1.4188	0.3608	2081
DN × St	2	8.6748	4.3374	9.0596	0.1067	9955
Si × St	2	4.9086	2.4543	0.37887	0.7041	9958
Ha × DN × Si	1	20.591	20.591	39.198	**0.0185**	7419
Ha × DN × St	2	2.6531	1.3265	2.5252	0.2824	9965
Ha × Si × St	2	6.3738	3.1869	0.49196	0.6367	9948
DN × Si × St	2	0.95753	0.47877	7.3906E-2	0.9353	9950
Ha × DN × Si × St	2	1.0506	0.52531	8.1091E-2	0.9282	9956
Residuals	120	777.36	6.478			

Ha = Habitat (i.e. cave/outside); DN = Day/night; Si = Sampling site; St = Sampling time. Significant p values are reported underlined and in bold. P(MC) = p values have been estimated by using Monte Carlo technique (i.e. perms < 200).

We observed 90 feeding events in the field. *A. imberbis* was observed eating in the water column and just over the bottom, always at night, with 93.4% of these events occurring outside the caves, mostly on rocky bottoms and *Posidonia* *oceanica* meadows, and to a lesser extent on pebbles.

## Discussion

Our first question was whether cave-dwelling *A. imberbis* mostly feeds at night. We found that the majority of stomachs were full when collected early in the morning and much less so when collected later in the day. In addition, we observed in the field that most (>90%) of the feeding events occurred at night and outside the caves. Although previous studies already suggested this prevalent nocturnal activity^48–50^, our study showed for the first time and specifically for cave-dwelling *A. imberbis*, that this fish is mostly active in foraging at night.

We have also questioned whether the prey items of the cardinal fish are associated with habitats located outside the caves. Our gut-content analyses showed a wide range of prey, most of them associated with habitats other than caves. Clear differences also emerged among the three caves investigated, which could suggest that *A. imberbis* feeds upon what is more available in the habitats outside each cave. *A. imberbis*, like other apogonids, could thus be a non-selective opportunistic predator. In coral reefs, apogonids usually consume a wide variety of organisms from both reef and non-reef habitats (e.g., open waters, sands and seagrasses^52,55^).

Consistently with the few previous studies available^34,49,54,56^, we found that cave dwelling *A. imberbis* mainly feeds upon crustaceans, especially the amphipods. Mediterranean marine caves are characterized by the occurrence of abundant swarms of mysids^13,31,32^. Should *A. imberbis* feed within the cave, we would have expected to find mysids in a greater number of stomachs. However, although significantly present in stomach contents, mysids do not represent the main prey item, while amphipods do. More importantly, none of the dominant cave-dwelling mysid taxa (*Hemimysis* spp., *Harmelinella mariannae*, *Siriella gracilipes*) were found in the stomachs of *A. imberbis*^32,34,57^. The specimens of *Siriella* sp. found in several stomachs (Table 1) belonged to an unidentified species other than *S. gracilipes*. Mysids found in *A. imberbis* stomachs are non-cave suprabenthic species swimming very close to the substrate^58^. The presence of polychaetes among the prey items of *A. imberbis* is consistent with the data reported by Rastorgueff *et al*.^34^. While Garnaud^49^ and Pinnegar and Polunin^56^ reported that the diet of *A. imberbis* is significantly composed of fishes, our results are in disagreement with this finding.

To our knowledge, only two studies reported data on *A*. *imberbis* gut contents identifying prey items at the genus or species level. The observations carried out by Garnaud^49^ were done on *A*. *imberbis* specimens caught by fishermen, with no information about the habitat type where they had been collected. Zupo and Stübing^54^ caught *A*. *imberbis* in *P. oceanica* meadows, so in a different habitat from caves. Parts of our results concerning the diet of *A*. *imberbis* agree with the two above-mentioned studies (e.g. the presence of decapods of the genus *Processa* and of euphausiids of the genus *Nychtiphanes*), but the determination of prey items at finer taxonomic level (species or genus) also enabled us to provide novel evidence on cave-dwelling *A*. *imberbis*. Taking into account the biology and ecology of the prey taxa we have identified, certain observations can be made concerning the feeding strategies of the cave-dwelling cardinal fish, with regard to its nocturnal feeding outside the caves. Feeding grounds seem to include open waters, rocky-algal reefs, sandy bottoms and seagrass meadows. In a preliminary study conducted in the Western Mediterranean (Corsica Island), Webster *et al*.^48^ observed *A. imberbis* foraging in both rocky reefs and *P*. *oceanica* beds at night. In our study, we consistently detected in the stomachs of the cardinal fish the presence of invertebrates commonly associated with phytal habitats (algae and seagrass). This is the case for the amphipods *Dexamine spinosa, Apherusa* spp. and *Hyale* spp.^59,60^, that altogether represented >50% of all amphipods in stomachs; the decapod *Galathea bolivari*^61,62^; and the polychaetes *Lysidice ninetta* and *L. unicornis*^63^. Many of the crustacean species identified usually remain hidden or burrowed within the substrate during the day and migrate to the water column at night^59,62,64,65^, where they could be more easily preyed upon by *A. imberbis*. As regards amphipods, for example, many of the most abundant species, such as *Dexamine spinosa*, *D. spiniventris, Apherusa* spp. or *Atylus vedlomensis*, could be sporadically hunted in the substrate, but their high abundance in the fish stomachs make them more likely to be picked up in the water column at night. A similar predation pattern has been observed for the non-Mediterranean *Apogon semilineatus* feeding on gammarids moving up to the near-bottom waters^66^. Some euphausiids found in the *A. imberbis* stomach contents, e.g. *Meganychtiphanes norvegica*, are typical of upwelling zones, such as the immediate vicinity of the Villefranche-sur-Mer Bay^67^. They belong to the migrating krill species that perform diel vertical migrations from shallow waters at night to greater depths during the day^68^. It is likely that when leaving the caves at night, *A. imberbis* can feed upon them in the water column. Regarding the polychaete prey, *Lysidice ninetta* and *L. unicornis* are quite common food items. Both species have a cryptic behaviour pattern, boring into calcareous algae and *P. oceanica*^62,69^. Their cryptic life style would therefore make them difficult to be preyed upon by *A. imberbis*. We found these polychaetes full of eggs in the fish stomachs, and most of them had eyes that were larger than usual, which are typical features of these worms during reproduction, when they become planktonic^70^. The two *Lysidice* species belong to the same family as the well-known ‘palolo worm’ (*Palola viridis*), whose reproduction involves mass spawning at night^69^. Studies conducted in the Mediterranean Sea suggest that *L. unicornis* reproduction takes place in March-June, earlier than *L. ninetta*, whose reproduction seems to occur in summer - late autumn^69^. From this perspective, we noted a good match between the reproduction periods of the two polychaetes and the sampling dates of the stomachs when we found these worms. It is thus very likely that *A. imberbis* feeds upon the two polychaetes during their planktonic phase of reproduction.

With regard to our last question, whether *A. imberbis* is more abundant in caves or outside during the night, our results based on day- and night-time visual censuses show that during the day, the density of *A. imberbis* is very high inside the caves and low outside. Densities recorded in the daytime outside the three investigated caves were similar to those reported in previous studies conducted in rocky reefs in the western Mediterranean Sea (e.g.^71,72^). At night, the pattern observed was the opposite, but with less pronounced ‘inside *vs*. outside’ differences. Only Azzurro *et al*.^71^ reported data on the cardinal fish censused by night in rocky reefs: density values are consistent with the data of this study. The present study is the first that has compared the density of *A. imberbis* between caves and rocky reefs outside, by day and by night, and the data suggest a migration of this fish at night, from the caves to external habitats.

Overall, the results concerning the three questions of this study are thus consistent with the hypothesis of the nycthemeral migration of *Apogon imberbis* inside/outside marine caves for feeding that could mitigate their trophic depletion (see^37^ and references therein). This evidence has significant implications for the trophic dynamics of marine caves: *A. imberbis* could contribute to a horizontal transfer of organic matter (not quantified yet), something that has been proved and calculated for *Hemimysis* mysids^31^. The faecal pellets released by *A. imberbis* would increase the cave trophic load, either directly or indirectly supporting higher biomasses of decomposer bacteria^73^. Cave mysids involved in horizontal organic matter transfer actually feed on quite different types of organic matter^34^ than *A. imberbis*, which is positioned much higher in the trophic web than mysids^34^. Fish and mysids could thus contribute to the diversification of the quality of organic matter made available to the cave ecosystem and transferred from the outside. This is a good example of a link between species and functional diversity.

## Conclusion

**Figure 6:**
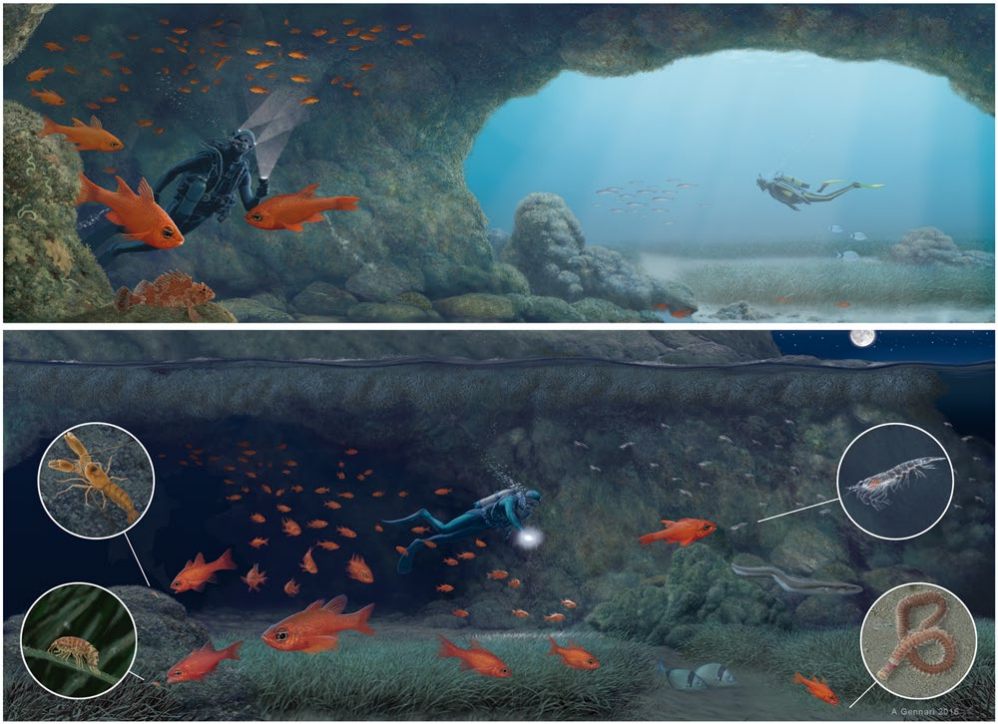
Hypothesized nycthemeral behaviour of cave-dwelling *Apogon imberbis*: Upper schema, diurnal activity; Lower schema, nocturnal activity. Concept by SB, A.D.F, PG. Illustrations by A. Gennari.

We have provided here new evidence regarding the potential of *A. imberbis* as a horizontal trophic vector in Mediterranean marine caves. The cave-dwelling *A. imberbis* stays within caves during the day, moves out during the night to then comes back into the caves, feeding at night on invertebrates associated with habitats other than caves (Fig. 6). This evidence supports the hypothesis that *A. imberbis* could contribute to enriching marine caves with POM, thus reducing the cave food depletion (see^37^ and reference therein).

The ecological relevance for biodiversity and the intrinsic fragility of marine caves in the Mediterranean Sea have been widely recognized^1^. Marine caves are thus listed among the European habitats requiring the designation of special areas of conservation (Annex I of the EU Habitat Directive^74^). Within this framework, a better understanding of their assemblages and their functioning, such as that resulting from this study, may surely provide a helpful basis for optimizing management and conservation measures^75,76^.

## Material and Methods

### Ethic statement

Sample collection was performed in strict accordance with the authorization protocol provided by Prefecture of the Provence-Alpes-Côte d’Azur Region (Permit Numbers: 715/2014 and 288/2015).

### Study sites

The study was conducted in three steps, designed to answer three specific interrelated questions aimed at testing the hypothesis regarding the ecological role of cave-dwelling *Apogon imberbis* as a potential organic matter vector in marine caves. The sampling effort thus varied depending on the phase of the investigation. First, we collected cardinal fish specimens during the period December 2014-August 2015 in 14 caves located in 7 areas along the coasts of the north-western Mediterranean Sea, from north-eastern Spain to western Italy: 3 caves at Cap de Creus (Spain), 3 at Côte Bleue, 3 at Villefranche-sur-Mer (France), 1 at the Principality of Monaco (Monaco), 1 at Ventimiglia, 1 at Bergeggi and 2 at Tavolara Island (Italy) (Fig. 2), to obtain information regarding their feeding patterns. For stomach content analyses of *A. imberbis*, we focused on 3 caves at Villefranche-sur-Mer (Fig. 2), while for the observation on nycthemeral distribution patterns we collected data in 2 of these 3 caves (Corail and Lido caves). The caves investigated encompassed blind-end caves (with one entrance) and caves with several openings, and were remarkably variable in morphology (e.g. branching), overall extent, presence/absence of air/chambers, and characteristics of the bottom (e.g. formed by rocks or sandy/muddy sediment), within the bathymetric range 3–24 m. Diver-scientists working on the project received specific training as marine caves typically contain fine sediments on the floor, which can easily be stirred up reducing visibility to zero and making it difficult if not impossible to locate the exit.

### Feeding patterns

The first question was: do cave-dwelling *A. imberbis* feed at night? If so, then stomach repletion should change according to a nycthemeral cycle. To highlight a possible day/night pattern in feeding, individuals were sampled at different times during the day (within a time period between 7:30 am and 5:00 pm). Individuals were collected in 14 caves from the 7 above-mentioned areas in the NW Mediterranean Sea (Fig. 2). For this phase of the study, we selected caves a few to hundreds of kilometres apart in order to be able to generalize our outcomes as much as possible.

Fish were collected using SCUBA and dip nets, and were preserved in ethanol after instant sacrifice through concussion of the brain by striking on the cranium (according to EU Recommendation 2007/526/EC) to minimize suffering. All specimens collected were measured (total length, to the nearest mm) and dissected to extract the stomachs. A total of 738 stomachs were dissected and analysed. Thirty-two stomachs were excluded as they belonged to mouth-brooding males, which do not feed while breeding eggs (their inclusion could have produced spurious indications on feeding patterns). We defined stomach state on a binary scale: empty (0) or full (1). We adopted this simple descriptor of feeding activity because a considerable number of prey were too small and/or too digested to be counted or weighed. We then performed a logit regression using the binary metric (empty/full stomach) as the outcome and the time-interval between sunrise and time of sampling (in hours) as the predictor. We selected this predictor in order to normalize our sampling time against the timing of sunrise, which was variable depending on the sampling day and site.

### Analysis of stomach contents

The second question was: do prey items of cave-dwelling *A. imberbis* belong to taxa typically associated with habitats outside the caves? In response to this question, the presence in the *A. imberbis* stomachs of species typically or exclusively associated with habitats other than caves (e.g. meadows of the seagrass *Posidonia oceanica*, rocky reefs or sandy substrates) would support the hypothesis that *A. imberbis* moves out of caves to feed at night. Fish were collected in the three caves located at Villefranche-sur-Mer (named ‘Corail’, ‘Lido’ and ‘Semaphore’ caves), where the external habitat is a mosaic of *P. oceanica* patches mixed with rocky and sandy substrates. Stomachs were analysed for the identification of prey items and a total of 184 specimens were collected and examined. All stomachs containing identifiable food items were used in the study. We counted individual prey items and expressed them as a numerical percentage over the total number of stomachs analysed. We pooled the data from the three caves to provide a general picture of *A. imberbis* diet composition. The most representative crustacean groups (i.e. amphipods, decapods, euphausiids, mysids) and the polychaetes found in the stomachs were dispatched among co-authors (i.e. experienced taxonomists familiar with cave fauna) for identification at the finest level and their frequency of occurrence in percentage (FO%) and mean number of individuals of identifiable prey items (N) in the stomachs of *A. imberbis* was calculated for each of the three caves. We formally tested the difference between caves using a one-way permutational multivariate analysis of variance (PERMANOVA)^77,78^ on square root transformed data with cave as random factor with 3 levels.

### Nycthemeral distribution pattern of *Apogon imberbis*

The third question was: are there changes in the abundance patterns of *A. imberbis* within and outside caves during the day/night cycle? To document whether *A. imberbis* moves outside marine caves at night to feed, we adopted a sampling design that included data collection on fish density both inside and outside caves, by day and by night. Sampling was carried out in 2 caves located at Villefranche-sur-Mer: the ‘Corail’ and ‘Lido’ Caves (factor Si = Sampling site). Density of *A. imberbis* was estimated using underwater visual census. Specifically, we used 25 m long and 2 m wide transects for sampling outside the caves^79^, and the modified transect visual census with fixed width (2 m) and variable length inside the caves^15^. Due to the variable area covered by each transect, fish density values were converted to number of individuals per 50 m^2^.

Visual censuses were conducted at 3 random dates during the period June–July 2015. On each of the three dates (factor St = Sampling time), we recorded density of the cardinal fish during diurnal (from noon to 2 pm) and night hours (from midnight to 2 am) (factor DN = Day/Night) at both sampling sites. At each sampling site, and for each date and day/night hours, visual censuses were performed both within and outside each cave (factor Ha = Habitat). Six replicate transects were carried out for each combination of factors, for a total of 144 replicates. We performed a PERMANOVA^77^ based on Euclidean distance measure of square root transformed density data using a sampling design that included 4 factors: sampling site (random, 2 levels), sampling date (random, 3 levels), day/night (fixed, 2 levels) and inside/outside habitat (fixed, 2 levels). All factors were orthogonal.

### Data availability

The datasets generated and/or analysed during the current study are available from the corresponding author on reasonable request.
